# Types of Obesity and Its Association with the Clustering of Cardiovascular Disease Risk Factors in Jilin Province of China

**DOI:** 10.3390/ijerph13070685

**Published:** 2016-07-07

**Authors:** Peng Zhang, Rui Wang, Chunshi Gao, Yuanyuan Song, Xin Lv, Lingling Jiang, Yaqin Yu, Yuhan Wang, Bo Li

**Affiliations:** Department of Epidemiology and Biostatistics, Jilin University School of Public Health, 1163 Xinmin Street, Changchun, Jilin 130021, China; peng14zhang@sina.com (P.Z.); ruiwang14@mails.jlu.edu.cn (R.W.); jlugcs@126.com (C.G.); songyuanmei00@163.com (Y.S.); jdhkl_lx@163.com (X.L.); jianglingling.2008@163.com (L.J.); pengzhang14@mails.jlu.edu.cn (Y.Y.); 8626699shiwo@163.com (Y.W.)

**Keywords:** obesity type, cardiovascular diseases, central obesity, compound obesity, clustering

## Abstract

Cardiovascular disease (CVD) has become a serious public health problem in recent years in China. Aggregation of CVD risk factors in one individual increases the risk of CVD and the risk increases substantially with each additional risk factor. This study aims to explore the relationship between the number of clustered CVD risk factors and different types of obesity. A multistage stratified random cluster sampling design was used in this population-based cross-sectional study in 2012. Information was collected by face to face interviews. One-way analysis of variance (ANOVA), chi-square test, Kruskal-Wallis test and multiple logistic regression were used in this study. The prevalence of general obesity, central obesity and compound obesity were 0.3%, 36.1% and 14.7%, respectively. The prevalence of hypertension, hyperlipidemia and diabetes in the compound obesity group were higher than those in other groups (compound obesity > central obesity > general obesity > non-obesity), while smoking rate in the non-obesity group was higher than those in other groups (non-obesity > general obesity > central obesity > compound obesity). People with obesity were more likely to have one or more CVD risk factor compared with non-obesity subjects (general obesity (OR: 2.27, 95% CI: 1.13–4.56), central obesity (OR: 2.64, 95% CI: 2.41–2.89), compound obesity (OR: 5.09, 95% CI: 4.38–5.90). The results were similar when the number of clustered CVD risk factors was ≥ 2 and ≥ 3. As a conclusion, more than half of the residents in Jilin Province have a problem of obesity, especially central obesity. Government and health department should take measures to improve people’s awareness of central obesity in Jilin Province of China. The prevalence of hypertension, hyperlipidemia and diabetes are associated with obesity types. Compound obesity has a greater risk to cluster multiple CVD risk factors than central obesity and general obesity. Taking measures to control obesity will reduce the prevalence of CVD in Jilin Province.

## 1. Introduction

Obesity is a medical condition in which body fat accumulates to a certain degree, which may have adverse effects on body, thereby reducing the life expectancy and health condition [1]. Evidence suggests that health impairments of obesity not only associate with the degree of obesity and fat content, but also relate to ectopic accumulation of body fat [2]. Central obesity is proven to be more suitable to explain obesity-related health risks when metabolic syndrome was taken as an outcome measure [3]. The differences among types of obesity need to be paid more attention.

Cardiovascular disease (CVD) is a class of diseases that involve the heart or blood vessels and is the leading cause of death globally, except in Africa [4]. Deaths caused by CVD have been increasing in many developing countries, while rates have declined in most of the developed countries since the 1970s [5]. China is the largest developing country in the world. The rate of increase of CVD in China was alarming and the related economic burden was estimated to be 550 billion dollars from 2005 to 2015 [6,7]. To our knowledge, the data of CVD and obesity in Jilin Province were insufficient before this investigation. Understanding the situation of CVD and obesity can provide useful information for formulating health policy. Hypertension, hyperlipidemia, diabetes, smoking and overweight are the five general accepted risk factors of CVD. Evidence suggests that a combination of these risk factors aggravates the risk of CVD and the risk will continue to grow with the increase of the number of clustered risk factors [8,9]. At present, a number of studies have been performed on the aggregation of CVD risk factors, but studies with regard to the influence of different types of obesity on the aggregation of CVD risk factors are relatively rare.

Jilin Province, with a population of about 27 million, is located in the northeast of China [10]. Data in our study were obtained from the Jilin Provincial Chronic Disease Survey in 2012, which is the first large representative population-based survey of chronic disease in this area. By exploring the relationship between CVD and different types of obesity, we hope to find an effective way to reduce the prevalence of CVD in Jilin Province. In our study, we explored the prevalence of different types of obesity; rates of CVD risk factors were also compared among different obesity groups. Adjusted association between the number of clustered CVD risk factors and different types of obesity was also examined in order to clarify the influence of different types of obesity on the aggregation of CVD risk factors.

## 2. Materials and Methods

### 2.1. Subjects

A population-based cross-sectional survey was conducted among residents who were 18–79 years old and were living in Jilin Province for over six months in 2012. A multistage stratified cluster sampling method was used to select the study sample. Nine regions (Changchun, Jilin, Siping, Liaoyuan, Tonghua, Baishan, Songyuan, Baicheng and Yanbian), 32 districts or counties, 95 towns or communities, and 45 units in Jilin Province were selected. Details of the stratification process were reported previously [11]. We recruited a total of 23,050 subjects and 21,435 completed the survey, which resulted in a response rate of 84.9%. Response rates of urban and rural areas were 81.8% and 88.6%, respectively. A total of 18,137 subjects with complete information of blood pressure, plasma glucose, serum lipids, body mass index (BMI) and waist circumference (WC) were chosen for the study.

### 2.2. Ethical Standards

Ethical approval was obtained by Jilin University School of Public Health, and written informed consent was obtained from all subjects. (Reference number: 2012-R-011).

### 2.3. Data Collection

The data of this survey is composed of three parts: questionnaire investigation (socio-demographic characteristics and health related information), body measurements (such as height, weight, waist circumference and blood pressure) and laboratory measurements (such as serum cholesterol and triglycerides). All investigators were trained and followed the same questionnaire instructions.

### 2.4. Measurements

The height of the subjects was measured to the nearest 0.1 cm without shoes using an anthropometer. Weight was measured in light clothing to the nearest 0.1 kg after removal of shoes. The measurement of height and weight were taken once. A calibrated mercury sphygmomanometer was used to determine the blood pressure of subjects on the right arm, after at least 5 min of seated rest. Blood pressure was measured three times with intervals of at least one minute, and we used the average value for data analysis. The investigator placed the tape 0.5–1.0 cm above the navel level around a circle to measure waist circumference. At the same time, the subjects were required to breathe naturally and wear thin clothes. The blood sample was obtained in the morning from subjects after fasting for at least eight hours, and then conserved in tubes which contained ethylenediaminetetraacetic acid (EDTA) [12].

### 2.5. Definitions 

According to the criteria of the “Chinese Guidelines on Prevention and Treatment of Dyslipidemia in Adults” [13], the diagnosis of hyperlipidemia is based on the presence of one or more of the following criteria: TC (total cholesterol) ≥ 5.18 mmol/L or TG (triglyceride) ≥ 1.70 mmol/L or HDL-C (high density lipoprotein cholesterol) < 1.04 mmol/L or LDL-C (low density lipoprotein-cholesterol) ≥ 3.37 mmol/L or previously diagnosed as hyperlipidemia by a physician. We defined diabetes as participants who reported diabetes mellitus previously diagnosed by aphysician or those who have fasting plasma glucose (FPG) ≥ 7.0 mmol/L or oral glucose tolerance test (OGTT) 2 h plasma glucose (PG) ≥ 11.1 mmol/L [14]. Self-reported hypertension and/or abnormal blood pressure (systolic ≥ 140 mmHg or diastolic ≥ 90 mmHg) were regarded as hypertension [15]. According to the criterion of weight for Chinese adults [16], BMI ≥ 28 was defined as obesity, and WC ≥ 80 cm for females, and WC ≥ 85 cm for males was defined as central obesity. In this study, we divided participants into four groups: non-obesity, general obesity, central obesity and compound obesity. Participants with normal WC but BMI ≥ 28 were defined as general obesity; participants with excessive WC only (WC ≥ 80 cm for female, WC ≥ 85 cm for male) were defined as central obesity; compound obesity was the combination of general obesity and central obesity; non-obesity was neither general obesity nor central obesity. Smoker was defined as a person who had smoked at least one cigarette per day in the past 30 days. Drinker was defined as a person who consumed more than one alcoholic drink per week, including any form of alcohol.

### 2.6. Data Analysis

Chi-square tests were used to compare the distribution of obesity types in different socio-demographic characteristics. One-way ANOVA or Kruskal-Wallis test were used to compare the level of CVD risk factors in different types of obesity. Rates of CVD risk factors in different obesity types were compared by chi-square test. The result of the trend test was analyzed by linear-by-linear association. Multiple logistic regression analyses were employed to adjust for potential confounding factors and explore the association between the number of clustered CVD risk factors and different types of obesity. *P* ≤ 0.05 was considered to be statistically significant. All the data analyses were conducted by SPSS 22.0 (IBM Corp, Armonk, NY, USA).

## 3. Results

This study included 18,137 subjects with complete information of blood pressure, plasma glucose, serum lipids, BMI and WC. Subjects ranged in age from 18 to 79 (mean ± SD: 47.64 ± 13.18). 8401 males (46.3%) and 9736 females (53.7%) were included in this study.

Table 1 describes the prevalence of different types of obesity. The prevalence of general obesity, central obesity and compound obesity were 0.3%, 36.1% and 14.7%, respectively. Among subjects with different types of obesity, the distribution of age, sex, occupation, family per capita monthly income and smoking are discrepant.

Table 2 describes the level of blood pressure, plasma glucose and serum lipids in different types of obesity. There are significant differences in the four groups of obesity.

The rate of CVD risk factors in different types of obesity are shown in Table 3. The prevalence of hypertension in non-obesity, general obesity, central obesity and compound obesity subjects is 22.7%, 44.2%, 47.2% and 58.2% respectively.

The prevalence of hyperlipidemia in non-obesity, general obesity, central obesity and compound obesity subjects is 41.9%, 50.0%, 69.6% and 76.5% respectively. The prevalence of diabetes in non-obesity, general obesity, central obesity and compound obesity subjects is 4.9%, 13.5%, 14.5% and 16.3% respectively. Smoking rates in non-obesity, general obesity, central obesity and compound obesity subjects are 32.6%, 23.1%, 28.9% and 26.1% respectively. Among subjects with different types of obesity, the prevalence of hypertension, hyperlipidemia, diabetes and smoking rates are different. The results of linear-by-linear association show that the prevalence of hypertension, hyperlipidemia and diabetes in the compound obesity group is higher than those in other groups (compound obesity > central obesity > general obesity > non-obesity), while smoking rate in the non-obesity group is higher than those in other groups (non-obesity > general obesity > central obesity > compound obesity). Table 4 describes the distribution of the number of CVD risk factors in different types of obesity. 34.4% of the non-obesity subjects have no CVD risk factor. 36.9% of the non-obesity subjects have one CVD risk factor. 36.5% of the general obesity subjects have one CVD risk factor. 30.8% of the general obesity subjects have two CVD risk factors. 31.9% of the central obesity subjects have one CVD risk factor. 36.7% of the central obesity subjects have two CVD risk factors. 28.4% of the compound obesity subjects have one CVD risk factor. 42.5% of the compound obesity subjects have two CVD risk factors.

Table 5 describes the association between the number of clustered CVD risk factors and different types of obesity. 65.6% of the non-obesity subjects have one or more CVD risk factors. 76.9% of the general obesity subjects have one or more CVD risk factors. 86.1% of the central obesity subjects have one or more CVD risk factors. 91.5% of the compound obesity subjects have one or more CVD risk factors.

**Figure 1:**
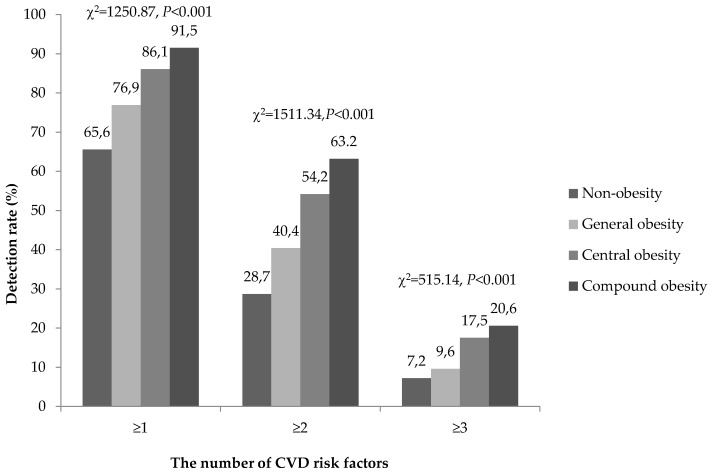
Detection rate of the number of clustered CVD risk factors in different types of obesity. Note: Linear-by-linear association.

Figure 1 shows that compound obesity group has the highest detection rate of one or more CVD risk factors (non-obesity < general obesity < central obesity < compound obesity). People with obesity were more likely to have one or more CVD risk factors compared with non-obesity subjects (general obesity (OR: 2.27, 95% CI: 1.13–4.56), central obesity (OR: 2.64, 95% CI: 2.41–2.89), compound obesity (OR: 5.09, 95% CI: 4.38–5.90)). The results were similar when the number of CVD risk factors was ≥ 2 and ≥ 3.

## 4. Discussion

To our knowledge, this is the first large population-based cross-sectional study to investigate the association between CVD risk factors and types of obesity in the Jilin Province of Northeast China. As described above, the prevalence of general obesity, central obesity and compound obesity were 0.3%, 36.1% and 14.7%, respectively. The overall obesity rate was 51.1%. The traditional definition of “obesity” was based on the criterion of BMI. Therefore, general obesity and compound obesity could be screened by BMI, but central obesity, which accounted for 36.1% of the total population, was easy to ignore. We suggest that the government and health department should take measures to improve people’s awareness of central obesity in the Jilin province of China. Employing two indicators of obesity would be a more efficient way to monitor residents’ health status. A study conducted in Japan showed that the rate of central obesity (central obesity + compound obesity in our study) was 50.7% in males and 20.8% in females [17]. Dewan’s study indicated that the rate of central obesity (central obesity + compound obesity in our study) in urban areas was higher than in rural areas (62.2% vs. 19.4%) [18]. Their results were different from ours. Gender and regional differences in the rates of central obesity (central obesity + compound obesity in our study) were not significant in our study (50.08% in malesvs. 51.49% in females; 50.69% in urban areasvs. 51.01% in rural areas). The possible explanation for this phenomenon may be due to different races and living habits.

Our results showed that the prevalence of hypertension, hyperlipidemia and diabetes in the compound obesity group were higher than those in other groups (compound obesity > central obesity > general obesity > non-obesity), while smoking rate in the non-obesity group was higher than that in other groups (non-obesity > general obesity > central obesity > compound obesity). Obesity increases the risk of many physical and mental conditions. These comorbidities are most commonly shown in metabolic syndrome [1], a combination of medical disorders which includes diabetes mellitus type 2, high blood pressure, high blood cholesterol, and high TG levels [19]. Some studies suggested that, to a greater extent, heart disease, hypertension, insulin resistance, and type 2 diabetes mellitus were linked with WC [20,21]. With the increase of overall WC, the risk of death also increased [22]. In a cohort study of the National Health and Nutrition Examination Survey (NHANES III), WC is proven to be more suitable to explain obesity-related health risks when metabolic syndrome was taken as an outcome measure [3]. In other words, excessive WC appears to be more of a risk factor for metabolic syndrome than BMI [23]. Our results indicated that the prevalence of hypertension, hyperlipidemia and diabetes were associated with obesity types. Central obesity was more dangerous than general obesity. When central obesity was compounded with general obesity, the risk of metabolic diseases would continue to grow. As many studies reported, tobacco use may affect the digestive and absorptive functions of the alimentary system [24,25]. Smokers were less likely to be obese compared with non-smokers.

Compound obesity was associated with greater risk to cluster multiple risk factors of CVD than central obesity and general obesity. Taking measures to control obesity, both in BMI and WC, would reduce the number of clustered risk factors of CVD and equally reduce the prevalence of CVD. Obesity is usually due to excessive intake of food energy and lack of physical activity. Physical activity assists weight loss and improves blood glucose control, blood pressure, lipid profile and insulin sensitivity. These effects may partly explain its cardiovascular benefits [4]. Dietary salt consumption is an important determinant factor of blood pressure level and overall cardiovascular risk [4]. High trans-fat intake has adverse effects on blood lipids and circulating inflammatory markers [26], and elimination of trans-fat from diets has been widely advocated [27]. Evidence suggests that higher consumption of sugar is associated with higher blood pressure and unfavorable blood lipids [28], and sugar intake also increases the risk of diabetes mellitus [29].

To sum up, this study showed that the prevalence of hypertension, hyperlipidemia and diabetes were associated with obesity types. The number of clustered CVD risk factors was also linked with obesity types. Different types of obesity have significant effect on the risk of CVD, and compound obesity has the highest risk of CVD (compound obesity > central obesity > general obesity > non-obesity). Therefore, taking measures to control obesity will help to reduce the prevalence of CVD in Jilin Province.

The strength of our study lies in a representative sampling survey based on a large population. However, the study still has limitations. First, our sample excluded those who were ill or too weak to complete the interview. Second, the participants were recruited from Jilin Province of China, so the conclusions cannot represent the situation in other regions of China. Third, the recall bias of all self-reported questionnaire interviews can’t be obviated.

## 5. Conclusions

More than half of the residents in Jilin Province have a problem of obesity, especially central obesity. The government and health departments should take measures to improve people’s awareness of central obesity in Jilin Province of China. The prevalence of hypertension, hyperlipidemia and diabetes were associated with obesity types. Compound obesity had a greater risk to cluster multiple CVD risk factors than central obesity and general obesity. Taking measures to control obesity will reduce the prevalence of CVD in Jilin Province.

## Figures and Tables

**Table 1:** Main characteristics of study population and the prevalence of obesity.

Characteristics	Non-Obesity *n* = 8863 (%)	General Obesity *n* = 52 (%)	Central Obesity *n* = 6542 (%)	Compound Obesity *n* = 2680 (%)	χ^2^	*p*
**Sex**					16.907	0.001
Men	4174 (47.1)	19 (36.5)	2913 (44.5)	1295 (48.3)		
Women	4689 (52.9)	33 (63.5)	3629 (55.5)	1385 (51.7)		
**Age**					847.342	<0.001
18~	4492 (50.7)	24 (46.2)	1851 (28.3)	989 (36.9)		
45~	3048 (34.4)	23 (44.2)	2968 (45.4)	1135 (42.3)		
60~	1323 (14.9)	5 (9.6)	1723 (26.3)	556 (20.8)		
**Area**					1.834	0.608
Urban	4625 (52.2)	27 (51.9)	3421 (52.3)	1362 (50.8)		
Rural	4238 (47.8)	25 (48.1)	3121 (47.7)	1318 (49.2)		
**Occupation**					339.090	<0.001
Manual	5383 (60.7)	24 (46.2)	3358 (51.3)	1415 (52.8)		
Intellectual	1865 (21.0)	16 (30.7)	1223 (18.7)	475 (17.7)		
Others	1615 (18.3)	12 (23.1)	1961 (30.0)	790 (29.5)		
**Family per capita monthly income (RMB)**					21.16	0.002
<1000	3645 (41.2)	20 (38.5)	2828 (43.2)	1164 (43.4)		
1000–3000	4382 (49.4)	24 (46.2)	3131 (47.9)	1225 (45.7)		
>3000	836 (9.4)	8 (15.3)	583 (8.9)	291 (10.9)		
**Smoking**					52.620	<0.001
Yes	2893 (32.6)	12 (23.1)	1893 (28.9)	699 (26.1)		
No	5970 (67.4)	40 (76.9)	4649 (71.1)	1981 (73.9)		
**Drinking**					4.793	0.188
Yes	1371 (15.5)	4 (7.7)	1013 (15.5)	383 (14.3)		
No	7492 (84.5)	48 (92.3)	5529 (84.5)	2297 (85.7)		

**Table 2:** Level of CVD risk factors in different types of obesity.

Risk Factor	Non-Obesity	General Obesity	Central Obesity	Compound Obesity	F/χ^2^	*p*
**Blood pressure (mmHg)**						
SBP	124.86 ± 19.26	130.77 ± 22.27	135.96 ± 21.34	140.31 ± 20.91	589.15	<0.001
DBP	76.65 ± 10.77	80.10 ± 11.87	81.93 ± 11.54	85.61 ± 11.81	551.11	<0.001
**Plasma glucose (mmol/L)**						
FPG (n = 16330)	4.9 (4.4, 5.4)	5.1 (4.6, 5.6)	5.2 (4.7, 5.9)	5.4 (4.8, 6.1)	782.85	<0.001
OGTT-2 h PG (n = 1807)	5.5 (4.8, 6.3)	5.8 (5.0, 6.8)	5.9 (5.1, 7.2)	6.1 (5.1, 7.4)	75.07	<0.001
**Serum lipids (mmol/L)**						
TC	4.56 (3.98, 5.22)	4.63 (3.90, 5.04)	5.00 (4.36, 5.71)	5.07 (4.45, 5.77)	863.73	<0.001
TG	1.14 (0.82, 1.66)	1.47 (0.99, 2.48)	1.83 (1.24, 2.75)	2.09 (1.46, 3.14)	2764.65	<0.001
HDL-C	1.45 (1.23, 1.73)	1.23 (1.04, 1.49)	1.26 (1.06, 1.49)	1.18 (1.00, 1.39)	1623.37	<0.001
LDL-C	2.66 (2.19, 3.23)	2.59 (2.19, 3.15)	3.05 (2.50, 3.66)	3.08 (2.52, 3.68)	808.79	<0.001

**Table 3:** Rate of CVD risk factors in different types of obesity.

Risk Factor	Non-Obesity n (%)	General Obesity n (%)	Central Obesity n (%)	Compound Obesity n (%)	χ^2^	*p*	*p*^a^
**Hypertension**					1588.21	<0.001	<0.001
Yes	2015 (22.7)	23 (44.2)	3088 (47.2)	1561 (58.2)			
No	6848 (77.3)	29 (55.8)	3454 (52.8)	1119 (41.8)			
**Hyperlipidemia**					1669.56	<0.001	<0.001
Yes	3710 (41.9)	26 (50.0)	4552 (69.6)	2051 (76.5)			
No	5153 (58.1)	26 (50.0)	1990 (30.4)	629 (23.5)			
**Diabetes**					519.63	<0.001	<0.001
Yes	433 (4.9)	7 (13.5)	947 (14.5)	437 (16.3)			
No	8430 (95.1)	45 (86.5)	5595 (85.5)	2243 (83.7)			
**Smoking**					52.620	<0.001	<0.001
Yes	2893 (32.6)	12 (23.1)	1893 (28.9)	699 (26.1)			
No	5970 (67.4)	40 (76.9)	4649 (71.1)	1981 (73.9)			

a: Linear-by-linear association.

**Table 4:** Distribution of the number of CVD risk factors in different types of obesity.

Number ofCVD Risk Factors	Non-Obesity n (%)	General Obesity n (%)	Central Obesity n (%)	Compound Obesity n (%)
0	3047 (34.4)	12 (23.1)	909 (13.9)	227 (8.5)
1	3271 (36.9)	19 (36.5)	2085 (31.9)	760 (28.4)
2	1906 (21.5)	16 (30.8)	2400 (36.7)	1142 (42.5)
3	588 (6.6)	3 (5.8)	997 (15.2)	500 (18.7)
4	51 (0.6)	2 (3.8)	151 (2.3)	51 (1.9)

**Table 5:** Association between the number of clustered CVD risk factors and different types of obesity.

Number of CVD Risk Factors	Type of Obesity	N (%)	Wald χ^2^	*p*	OR (95% CI) ^a^
≥1					
	Non-obesity	5816 (65.6)	–	–	1.00
	General obesity	40 (76.9)	5.27	0.022	2.27 (1.13, 4.56)
	Central obesity	5633 (86.1)	449.19	<0.001	2.64 (2.41, 2.89)
	Compound obesity	2453 (91.5)	457.89	<0.001	5.09 (4.38, 5.90)
≥2					
	Non-obesity	2545 (28.7)	–	–	1.00
	General obesity	21 (40.4)	5.59	0.018	2.08 (1.13, 3.81)
	Central obesity	3548 (54.2)	712.27	<0.001	2.70 (2.51, 2.90)
	Compound obesity	1693 (63.2)	876.62	<0.001	4.35 (3.95, 4.79)
≥3					
	Non-obesity	639 (7.2)	–	–	1.00
	General obesity	5 (9.6)	0.78	0.377	1.54 (0.59, 4.00)
	Central obesity	1148 (17.5)	289.28	<0.001	2.53 (2.28, 2.82)
	Compound obesity	551 (20.6)	333.68	<0.001	3.31 (2.91, 3.76)

Note: OR: odds ratio; CI: confidence interval; a: Adjusted for age, sex, occupation and family per capita monthly income.
